# Unlocking the potential of *Trichoderma harzianum* isolate Zag-1 to boost drought tolerance in maize plants

**DOI:** 10.1186/s12870-026-08410-3

**Published:** 2026-03-17

**Authors:** Rabab A. Metwally, Asmaa S. Taha, Shereen A. Soliman

**Affiliations:** Botany and Microbiology Department, Faculty of Science, Zagazig University, Zagazig, 44519 Egypt

**Keywords:** Drought, indole-3-acetic acid, Plant growth attributes, Osmoprotectant, *Trichoderma harzianum*

## Abstract

Drought stress is main abiotic constraint restrictive maize (*Zea mays* L.) growth worldwide. An effective agricultural approach to enhance its growth under this stress is the plant growth-promoting microorganism’s application. In this study, *Trichoderma harzianum* isolate Zag-1 was evaluated for its potential to mitigate drought-induced damage in maize plants. At first, *T. harzianum* was isolated from pepper plant’s rhizosphere soils, and its growth-promoting characteristics were assessed, including indole-3-acetic acid (IAA), hydrolytic enzymes (chitinase, amylase, and cellulase), ammonia production, and zinc and phosphorus solubilization, all of which showed positive results, confirming its growth-promoting attributes. Besides, an in vivo experiment was conducted under controlled greenhouse conditions by withholding irrigation until soil moisture dropped to ~ 45% field water capacity (FWC) for 15 days. Four treatments are used: (1) well-watered control (90% field water capacity, FWC), (2) *T. harzianum*-inoculated under well-watered conditions, (3) drought-stressed (45% FWC), and (4) *T. harzianum-*inoculated+ drought-stressed. After 15 days of drought application, morphological (shoot height, in addition shoot and root fresh and dry weights), physiological (Chl a and b, relative water content [RWC], and membrane stability index [MSI]) and biochemical (osmolytes [proline, proteins and total soluble sugars] and antioxidant enzyme activities) assessments were recorded. The results indicated that shoot (39.44 and 37.92%) and root (10.29 and 28.77%) fwt and dwt, leaf RWC (7.90%), total pigment content (24.49%) and MSI (9.40%) decreased in drought-stressed maize while an increase in their membrane lipid peroxidation (77.21%), electrolyte leakage (EL) (48.04%) and hydrogen peroxide (128.94%) contents were noted versus the control plants; however, *T. harzianum*-treated plants showed the reverse responses. *T. harzianum*-treated maize plants revealed significant boosts in growth and physiology under stress paralleled to untreated ones, along with a notable enhancement in antioxidant defense activity (21.20 and 8.53% for POD and APX). Moreover, under stress, osmoprotectants, including proline (23.78%) and soluble sugars (12.28%) were markedly greater in *Trichoderma*- inoculated plants than non-inoculated. Our results recommend that *T. harzianum* is an effective, sustainable biotechnological tool for drought resilience in maize. Therefore, exploring and optimizing the application of *T. harzianum* addresses the broader goals of resource recycling, environmental sustainability, and climate-smart agriculture.

## Highlights

Drought stress (45% field water capacity, FWC) considerably impaired maize growth and photosynthetic pigments (Chl a, Chl b, as well as carotenoids).

Relative water content and membrane stability index of maize dropped under drought, indicating severe dehydration and osmotic imbalance. However, T. harzianum application reverses these detrimental effects on morphology and physiology.

Under drought, stress markers (H2O2 and MDA, membrane injury in addition to electrolyte leakage) increased, confirming enhanced lipid peroxidation and cellular damage.

*T. harzianum* inoculation enhanced osmolyte levels (proline, total soluble proteins and sugars), assisting osmotic adjustment.

*T. harzianum* application upregulated antioxidant enzymes (APX, PPO, and POD), alleviating oxidative stress and improving drought tolerance.

## Introduction

Drought stress is a foremost abiotic restraint controlling global crop productivity besides sustainability, particularly under the cumulative impact of climate change. It arises when water availability falls below the plant’s requirements due to irregular precipitation, elevated temperatures, increased evapotranspiration, or reduced soil water-holding capacity [1–3]. The severity and duration of drought episodes have been intensifying in recent years, making it one of the leading threats to agricultural systems in arid as well as semi-arid regions worldwide [3–5]. Its effects are adversely impacting plant morpho-physiological, as well as biochemical processes [6]. Morphologically, drought reduces plant height, leaf expansion, root growth, and overall biomass, while promoting leaf rolling, early senescence, and abscission to minimize water loss [7–9]. At the physiological level, drought impairs photosynthesis due to stomatal closure, which restricts CO_2_ uptake, along with reduced Chl. content and disturbed electron transport in the photosystems [10]. Moreover, drought triggers oxidative stress due to excessive reactive oxygen species (ROS) accumulation that can harm lipids, proteins, and DNA, causing cellular injury [9, 11]. In response, plants trigger antioxidant enzymes such as ascorbate peroxidase (APX), catalase (CAT), and peroxidase (POD), accompanied by osmolytes buildup like proline, soluble sugars, and sugars to sustain cell turgor besides safeguard cellular structures [12, 13].

Understanding the complicated effects of drought is essential for developing sustainable solutions that are urgently desirable to enhance crop resilience. Among the most promising strategies is the beneficial fungi, particularly species from the genus *Trichoderma*, which are extensively recognized for their role as plant growth-promoting fungi (PGPF) as well as biocontrol agents [6, 14–17]. *Trichoderma* spp. enhance plant growth as well as stress tolerance *via* a diversity of mechanisms, such as the phytohormones production (e.g., indole-3-acetic acid), improvement of root architecture, increasing relative water content, Chl retention, enhancing nutrient solubilization, as well as plant antioxidant systems activation [18–20].

Under drought conditions, *Trichoderma* sp. can modulate the drought-responsive genes expression, increase proline and soluble sugar content, besides boost the antioxidant enzyme activities, averting oxidative damage, helping plants maintain cellular homeostasis, and lessening drought stress in a number of crops [6, 21]. It has been observed that *T. harzianum*-injected plants retain the activity of the plant growth regulators i.e.: IAA, IBA (indole butyric acid), and GA (gibberellic acid) during stress [22, 23]. Moreover, *Trichoderma* promotes drought adaptation by enhancing stomatal regulation and boosting osmotic adjustment *via* the compatible solutes accumulation [9, 24]. According to Gutiérrez-Chávez et al. [25], *T. asperellum* enhanced hydroponic lettuce’s growth and stress-related qualitative attributes. Under polyethylene glycol (PEG)-induced drought, barley seed-biopriming using *T. harzianum* revealed improved biomass, osmotic control, elevated IAA, and greater antioxidant enzyme activity [6].

Maize (*Zea mays* L.) is a key crop in Egypt, sustains livelihoods and export revenue in major producing nations, provides animal feed and staple foods, and serves as a basis for sizable ethanol and starch industries [26]. However, drought results in extensive yield losses [27, 28]. Global maize yields are still closely linked to climate variability and drought exposure because technological advancements and high input use have recently driven national yields to record levels in some regions, while increased heat and more frequent seasonal dryness have already reduced achievable gains and caused regional declines in others [29].

Recent studies have highlighted the beneficial role of *Trichoderma* species in mitigating stresses, through enhancement of plant growth, antioxidant defence, and osmolyte accumulation. However, drought tolerance efficiency can differ among strains depending on their intrinsic stress-adaptive traits. In this study, we focused on *T. harzianum* isolate Zag-1, which exhibits distinct physiological and biochemical characteristics associated with stress resilience. Based on these observations, we hypothesize that Zag-1 may provide superior drought-alleviating effects, making it a promising candidate for sustainable and climate-smart agricultural applications. To test this hypothesis, we evaluated its effects on the morphological, physiological, and biochemical responses of maize under drought stress, aiming to assess its potential as a biological tool for enhancing crop resilience in climate-smart agriculture.

## Materials and methods

### Fungal strain and maintenance

*Trichoderma harzianum* isolate Zag-1 was originally attained from rhizosphere soil samples from fields of Sahragat El-Kobra, Mit Ghamr, Dakahlia, cultivated with pepper (*Capsicum annuum* L.) plants. The isolate was purified and molecularly identified based on the internal transcribed spacer (ITS) region sequencing, and the sequence was subsequently placed in the NCBI GenBank with the accession number [PX649202]. The strain was routinely preserved on Potato Dextrose Agar (PDA) [Sigma-Aldrich, St. Louis, MO, USA] at 25 ± 2 °C and sub-cultured at 14-day intervals to preserve its viability.

### Plant Growth-Promoting (PGP) traits

#### Assessing extracellular enzymatic potential in *T. harzianum*

*T. harzianum’s* chitinase enzyme was detected on 1% colloidal chitin agar medium. After 1 week of incubation, the plates were stained with Congo red for 1 h (0.1%) and then destained with NaCl (1 M). Chitin hydrolysis was recognized by the clear zone formation around the colony [30].

*T. harzianum’s* amylase activity was assessed by growing it on starch (0.2% soluble starch) agar for 7 days at 28 °C. After incubation, plates were flooded with iodine solution and the development of a clear zone suggested enzymatic starch hydrolysis [31].

Cellulase activity was evaluated on agar medium containing carboxymethyl cellulose (CMC, 0.5%) [32]. After incubation (7 days) at 28 °C, *T. harzianum* plates were stained with Congo red and destained with NaCl (1 M). Cellulose breakdown was detected by orange or transparent halos [33].

#### Phosphorus and zinc solubilization

*T. harzianum* was spotted on Pikovskaya’s plates and incubated at 25 ± 2 °C for 5 days. Formation of a clear halo around the fungal colony was considered positive for solubilization [34]. Also, its ability to solubilize insoluble Zn was investigated using Tris-minimal agar media supplemented with 0.1% zinc oxide (ZnO). Spot inoculation was done, and plates were incubated for 5 days at 25 ± 2 °C. The formation of distinct halos around colonies directed positive Zn solubilization.

#### Ammonia production

*T. harzianum* was tested for its ability to produce ammonia [35]. It was grown in 25 mL of sterilized peptone water and incubated at 25 ± 2 °C for 1 week. After incubation, the supernatant (1 mL) was added to 1 mL of Nessler’s reagent and the formation of a brown to yellow color represented a positive result [36].

#### Indole-3-Acetic Acid (IAA)

*T. harzianum* Zag-1 was inoculated in PDB media supplemented with L-tryptophan (0.5 g/L) to assess IAA production. It was incubated at 25 ± 2 °C in a shaker (100 rpm) for 5 days [37]. After that, the culture was centrifuged (10,000 rpm) for 10 min. Two mL of Salkowski’s reagent (1 mL of 0.5 M FeCl_3_ in 50 mL of perchloric acid [35%]) was mixed with supernatant (1 mL).

### Screening for *T. harzianum's* potential to tolerate drought stress

*T. harzianum* fungal discs (5 mm in diam.) were cultivated in 250 mL Erlenmeyer flasks enclosing 100 mL of PDB (potato dextrose broth media) having varying polyethylene glycol (PEG 6000 Da, Sigma Aldrich) concentrations to induce specific water potentials. Following the calculations established by Michel and Kaufmann [38], the concentrations used were 5% (-0.05 MPa), 15% (-0.31 MPa), 25% (-0.82 MPa), 35% (-1.68 MPa), 45% (-2.95 MPa), and 55% (-4.65 MPa). PDB media without PEG (0%; 0 MPa) served as the control. All flasks were incubated on a shaker (100 rpm) for 7 days at 25 ± 2 °C. After that, the fungal mycelia were filtered through sterilized filters and washed with dist. water, dried for 2 days at 60 °C, and weighed on pre-weighed Whatman no. 1 filter paper to determine their dry weight (dwt). The growth reduction in PEG-amended media was determined by the following formula.

$$\text{Growth reduction}\;\left(\%\right)\;=\;\frac{\mathbf{A}-\mathbf{B}}{\mathbf{A}}\;\times\mathbf{100}$$

A is the dry weight (dwt, g) of *T. harzianum* in the control flask, and B is the dwt in the drought-amended flask

### In vivo impact of *T. harzianum* Zag-1 on maize under drought stress

#### Plant material, experimental design, microbial inoculation, and growth conditions

To investigate the effects of drought stress and *T. harzianum* application on maize (*Zea mays* L.), a pot experiment was directed. Grains of drought-sensitive maize (Trihybrid 321, acquired from Agricultural Research Center, Giza, Egypt) were surface sterilized with ethanol (70%) for 1 min, afterward 2% sodium hypochlorite for 5 min and rinsed with sterile distilled water. Grains were sown in pots (30 cm diam.) full with 3 kg of sterilized clay soil and sustained in a greenhouse under natural photoperiod (25–30 °C, relative humidity 60–70%). The spore suspension of *T. harzianum* was diluted with water to 1 × 10^7^ cfu/mL after being cultivated for 7 days at 25 ± 2 °C on PDA medium and used to inoculate half of the pots in the sowing day as a soil drench (100 mL fungal spore suspension). The experimental design was a completely randomized design (CRD) using 5 replicates. After 10 days of sowing, an excess of 25 mL of *T. harzianum* spore suspension was added to each of the *T. harzianum-*treated maize seedlings. Drought stress was imposed after 15 days of sowing by withholding irrigation until soil moisture dropped to ~ 45% field water capacity (FWC) for 15 days, while the control was maintained at 90% FWC. Four treatments were included as follows:

1. Control – (90% FWC), no inoculation
2. Tricho – Well-watered+ T. harzianum-treated seedlings.
3. Drought Stress – Drought (45% FWC), no fungal inoculation.
4. Drought + Tricho – Drought + T. harzianum-treated seedlings.

### Measurements

#### Plant morphological indicators

At 15th day of drought application, maize plants were harvested and appraised for the morphological parameters. Shoot height (cm) was measured from the base to the highest point of the plant. Also, maize roots were carefully washed, and their lengths were measured. Leaf area of maize was calculated [39] according the following equation:

$$\text{Leaf area}\;\left(\mathrm{cm}^{2}\right)\;=\mathrm{length}\;\times\;\text{maximum width}\;\times\;0.75\;\left(\text{correction factor}\right)$$

The maize shoots and roots were assessed to record their fresh weight (fwt, g), then oven-dried at 70 °C for 72 h to obtain their dry weights (dwt, g).

#### Physiological assessments

Relative water content (RWC) as well as water saturation deficit (WSD) of maize leaves were considered [40].

$$\:\mathrm{R}\mathrm{W}\mathrm{C}\:\left(\%\right)\:=\frac{\mathrm{f}\mathrm{w}\mathrm{t}-\mathrm{d}\mathrm{w}\mathrm{t}}{\mathrm{t}\mathrm{w}\mathrm{t}-\mathrm{d}\mathrm{w}\mathrm{t}\:}\:\times\:100\\\mathrm{W}\mathrm{S}\mathrm{D}\:\left(\%\right)=\:100-\mathrm{R}\mathrm{W}\mathrm{C}$$

*fwt = fresh weight, twt = turgid weight after 24 h hydration, and dwt = dry weight.

After 15 days of drought exposure, the Chl content of maize leaves was quantified after 85% acetone extraction [41] and estimated spectrophotometrically at 663, 452.5 and 645 nm *via* a UV-visible spectrophotometer (RIGOL, Model Ultra-3660), conferring to the following formulae provided by Lichtenthaler and Wellburn [42].

$$\begin{aligned} &\text{Chl. a content}=12.7\left(\mathrm{A}_{663}\right)-2.69\left(\mathrm{A}_{644}\right)\times\mathrm{E.V}/\left(1000\times\mathrm{fwt}\right)\\&\text{Chl. b content}=22.9\left(\mathrm{A}_{644}\right)-4.68\left(\mathrm{A}_{663}\right)\times\mathrm{E.V}/\left(1000\times\mathrm{fwt}\right)\\&\mathrm{Carotenoids}=\left(4.2\;\mathrm{A}_{452.5}\right)-\left(0.0264\;\mathrm{Chl.a}\;+\;0.426\;\text{Chl. b}\right)\\& \quad \times\mathrm{E.V}/\left(1000\;\times\mathrm{fwt}\right) \end{aligned}$$

* E.V = Extraction volume of sample. 

#### Biochemical parameters

##### Stress indicators

Electrolyte leakage [EL] assessed the membrane damage caused by drought stress in leaves [43]. Deionized water (30 mL) was added to glass bottles that contained 0.5 g of chopped maize leaves that had been cut into tiny (10 mm) segments. After being left at ambient temperature for 24 h to detect the solution’s initial electrical conductivity (EC1), then the bottles were kept at 95 °C for 30 min to measure the solution’s final EC2. Additionally, the membrane stability index (MSI) was appraised. The membrane injury (MI) was considered [44] and derived from MSI by comparing the MSI of drought-affected maize plants to that of the controls. The equations of EL and MSI were defined as follows:

$$\mathrm{EL}\;\left(\%\right)=\mathrm{EC1}/\;\left(\mathrm{EC2}\right)\;\times100\\\mathrm{MSI}\;\left(\%\right)=\;1=\left(\mathrm{EC1/EC2}\right)\times100$$

Hydrogen peroxide (H2O2) content in 0.5 g of maize fresh leaves was determined at 390 nm next homogenizing with 5 mL of ice-cold 0.1% of trichloroacetic acid (TCA) [45]. After that, the supernatant (0.5 mL) was mixed with 0.5 mL of K-phosphate buffer (10 mM, pH 7.0) and 1 mL of potassium iodide (KI, 1 M) and incubated at room temperature for 1 h.

Lipid peroxidation was quantified through measuring malondialdehyde (MDA) by the thiobarbituric acid (TBA) [46]. Maize fresh leaves (0.5 g) were homogenized in 5 mL of TCA (0.1%), and 1 mL of the supernatant was mixed with 4 mL of TBA (0.5%) in 20% TCA. The mixture was put in a water bath (95 °C) for 30 minutes and then quickly cooled in ice. Samples were centrifuged again, and the absorbance was noted at 532 nm and then at 600 nm for nonspecific absorbance.

$$\mathrm{MDA}\;\left(\mathrm{nmol}\;g^{-1}\;\mathrm{fwt}\right)\;=\frac{A_{532}-A_{600}}{\text{Extinction coefficient of}\;155mM^{-1}cm^{-1}}\times\;10^{6}$$

#### Assessment of osmoprotectants content

Proline content in maize leaves was assessed at 520 nm [47] using acid ninhydrin and expressed as µmol g⁻¹ fwt. Total soluble sugars in dried (0.1 g) maize leaves were determined *via* the phenol–sulfuric acid method [48]. Absorbance measured at 490 nm. Total soluble proteins in maize fresh leaves (0.5 g) were assessed [49].

#### Assessment of antioxidant enzyme activities

Peroxidase (POD) was measured using guaiacol as a substrate at 470 nm. In a reaction mixture (1 mL) involving enzyme extract, ascorbate (5 mM), phosphate buffer (100 mM, pH 7.0), and H_2_O_2_ (0.5 mM), the ascorbate peroxidase (APX) activity was determined at 290 nm [50]. Also, polyphenol oxidase (PPO) activities were measured at 430 nm [51]. The enzyme activities were expressed as U/g fwt.

### Statistical analysis

All data were analyzed exhausting one-way ANOVA (Version 16.0, SPSS Inc., Chicago, IL, USA). Means were compared using Duncan’s multiple range test (DMRT, *p* ≤ 0.05) [52]. OriginPro 8.5 was used for data processing and graphing tools in the assembly of the figures.

## Results and discussion

Drought poses a serious danger to world agriculture since it stress disrupts plant development, photosynthesis, respiration, and hormonal balance [3, 6, 53]. Accordingly, one potential technique for boosting plant resistance in the face of water scarcity is the application of beneficial microorganisms like *T. harzianum* [54].

### In vitro assessment of PGP traits of *T. harzianum*

**Fig. 1:**
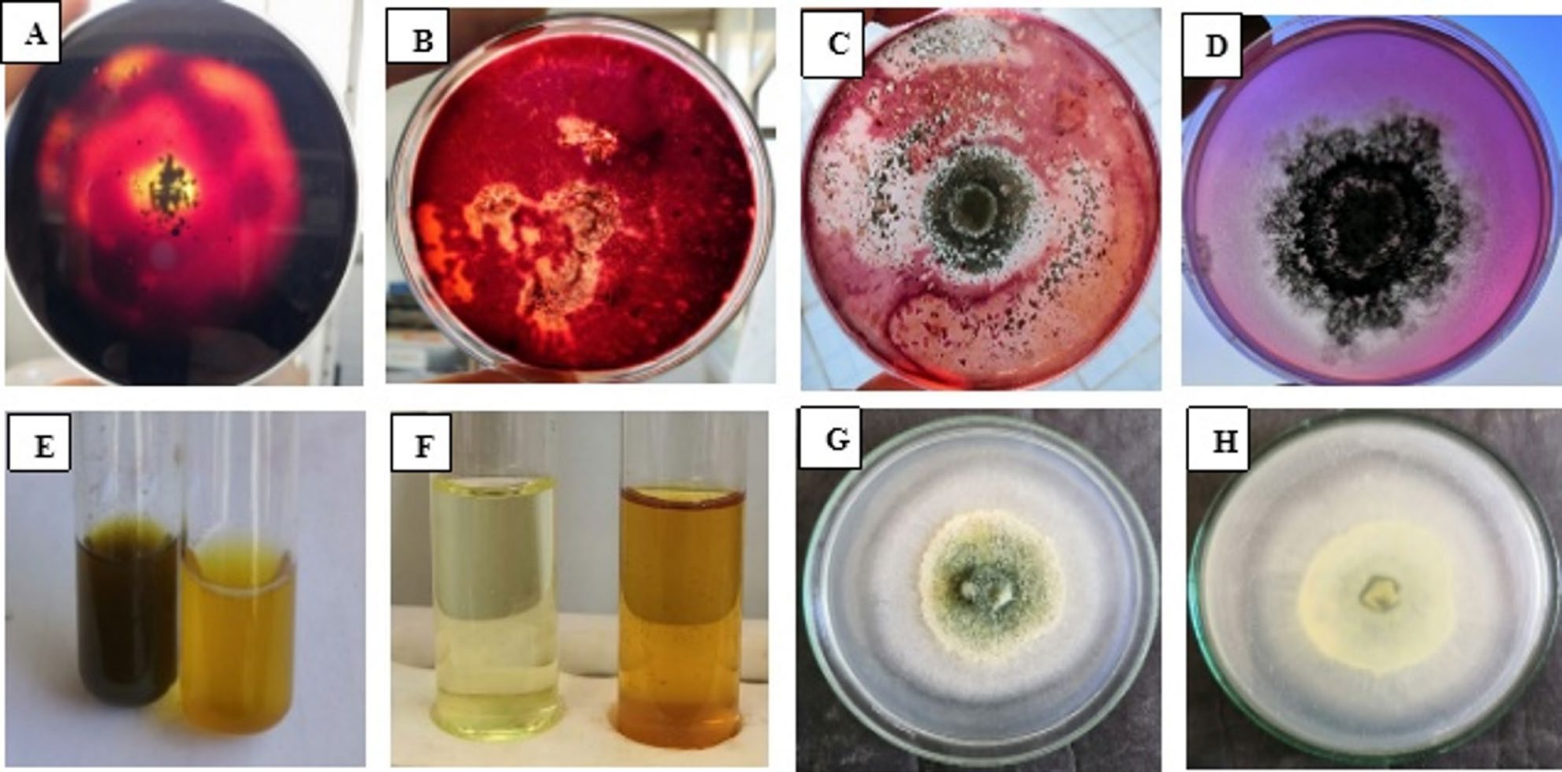
In vitro growth-promoting attributes of *T. harzianum* Zag-1: **A** Amylase activity [a clear halo on iodine-stained starch-amended medium], (**B**) Cellulose activity [an orange-pink halos around the growth on Congo red- stained CMC agar], (**C**) chitinase activity [a clear halo around the growth on Congo red- stained 1% colloidal chitin agar medium], **D** Phosphate solubilization [a clear halo zone around *Trichoderma* colonies on Pikovskaya’s medium], **E** Ammonia production [a color change from yellow to dark brown in inoculated peptone water upon Nessler’s reagent addition**]**, **F** IAA production [a dark yellow color development] and Zn solubilization [appearance of clear halo zone around colonies] from the front (**G**) and reverse (**H**) sides

The enzymatic analysis of *T. harzianum* Zag-1 indicated vital activity in all established extracellular enzymes, as clearly demonstrated in Fig. 1 (A–C). Amylase in *T. harzianum* was reported **(**Fig. 1A**)**, approving starch hydrolysis. Also, the cellulase activity was detected as an orange- pink halo around the growth on Congo red- stained CMC agar as appeared in Fig. 1 (B) and the same was for the chitinase activity in *T. harzianum* on colloidal chitin plates (Fig. 1C). Clear halo production on respective media indicated the presence and functionality of amylase, cellulase, and chitinase- key enzymes involved in the breakdown of complex polysaccharides in both pathogen cell walls as well as soil organic matter [19, 55]. Collectively, these findings highlight its ability to improve soil nutrient dynamics, endorse plant growth, and overcome phytopathogens by enzymatic degradation pathways. These aspects emphasize its possible in organic matter turnover, biopolymer degradation, and adaptive resilience within plant-associated ecosystems. As well, *T. harzianum* acts as a potent microbial inoculant in integrated nutrient and pest management systems.

The in vitro evaluation of *T. harzianum* Zag-1 shown multiple PGP attributes **(**Fig. 1D–H**)**. Figure 1 (D) show the ability of *T. harzianum* isolate Zag-1 to solubilize phosphate reflecting *T. harzianum’s* ability for enhance plant growth. Our results are in agreement with Bononi et al. [56], who demonstrated the potential of *Trichoderma* spp. for phosphate solubilization and increased plant growth. Figure 1 (E) show the ability of *T. harzianum* isolate Zag-1 to produce ammonia that enhance N_2_ content and root proliferation. Similar results were observed by Abdenaceur et al. [57] and Alwadai et al. [58], who reported ammonia production by *Trichoderma* spp., *T. koniniopsis*,* T. harzianum* and *T. velutinum* that can enhance N_2_ content in plants. Moreover, Fig. 1 confirmed IAA production Fig. 1 (F) and Zn solubilization of *T. harzianum* grown on ZnO medium photographed from the front Fig. 1(G) and reverse Fig. 1(H) sides as P and Zn are essential micronutrients involved in enzyme function, protein synthesis, and osmoregulation. These results are in line with Vinale et al. [59] findings that *Trichoderma* spp. secretes auxin-like compounds that positively impact plant root architecture. IAA is a key phytohormone involved in root initiation, cell elongation, and overall plant development [19]. *T. harzianum*’s ability to solubilize these nutrients can enhance their bioavailability in soils with limited mobility, aligning with findings by Hugar and Nayaka [19], who highlighted nutrient solubilization as a vital trait of PGP fungi.

### *T. harzianum’s* potential for drought stress tolerance

**Fig. 2:**
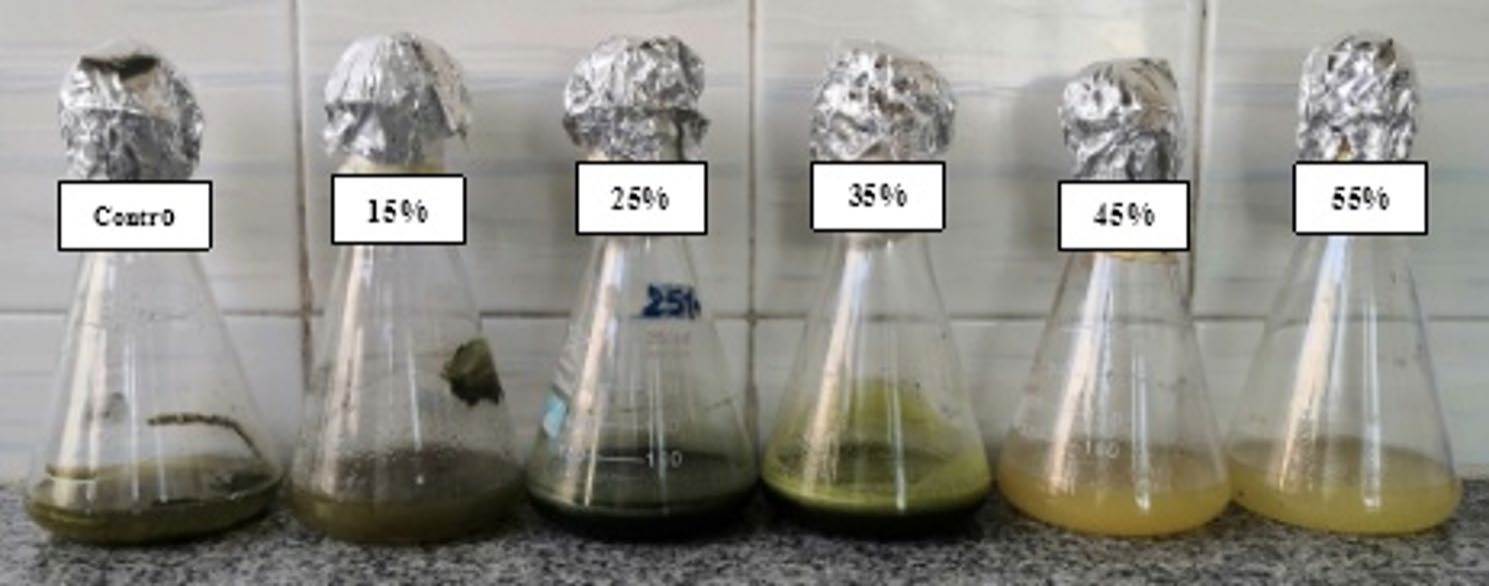
Growth pattern of *T. harzianum* Zag-1 growing in PDB supplemented with increasing levels of PEG (0–55%) under drought stress. The data are mean ± standard deviation from three replicates

The purpose of incorporating PEG into growing media is to gauge how water potential affects fungal growth [60]. There was a noticeable concentration-dependent decrease in the mycelial dwt of *T. harzianum* isolate Zag-1 grown in PDB amended with increasing PEG concentrations **(**Table 1 and Fig. 2**)**. The highest dwt was produced by cultures in un-amended PDB (0% PEG), while low-to-moderate PEG levels (15–25%) resulted in a slight but notable decrease in dwt and postponed biomass accumulation (Table 1). The reduction became more noticeable as PEG concentration increased, with mycelial dwt being markedly suppressed at high PEG (35–45%) levels. The reduction in medium water potential that restricts hyphal water uptake and turgor-driven expansion, which lowers growth rate and biomass accumulation, is the best explanation for the decline in mycelial dwt that occurs with increasing PEG concentrations. Meanwhile, osmotic challenge frequently rewires fungal metabolism (osmolyte accumulation and antioxidant induction), which can sustain survival but not biomass yield under extreme stress. Our findings support those of Rawal et al. [61], who found that all *Trichoderma* isolates’ mycelial growth declined as PEG increased. Therefore, using biological agents such as water deficit-tolerant *Trichoderma* may be a sustainable, affordable, and eco-friendly short-term way to lessen stress [62, 63].

**Table 1:** Impact of different polyethylene glycol (PEG 6000 Da) on *T. harzianum* Zag-1 mycelial dry weight (g/100 mL)

PEG conc.	Mycelial dry weight (g/100 mL)	Growth reduction (%)
0%	1.0195 ± 0.0269a	0 g
5%	0.966 ± 0.0255ab	5.247 ± 0.138f
15%	0.9103 ± 0.0240b	10.711 ± 0.283e
25%	0.8038 ± 0.0212c	21.157 ± 0.559d
35%	0.6802 ± 0.0179d	33.281 ± 0.880c
45%	0.2157 ± 0.0057e	78.842 ± 2.085b
55%	0f	100 ± 0.00a

Data represents the mean of 5 replicates with standard error. Different letters indicate significant differences among treatments using a one-way ANOVA followed by the Duncan’s multiple range test (*p* < 0.05)

### In vitro enhancement of maize growth under drought by *T. harzianum* Zag-1

**Fig. 3:**
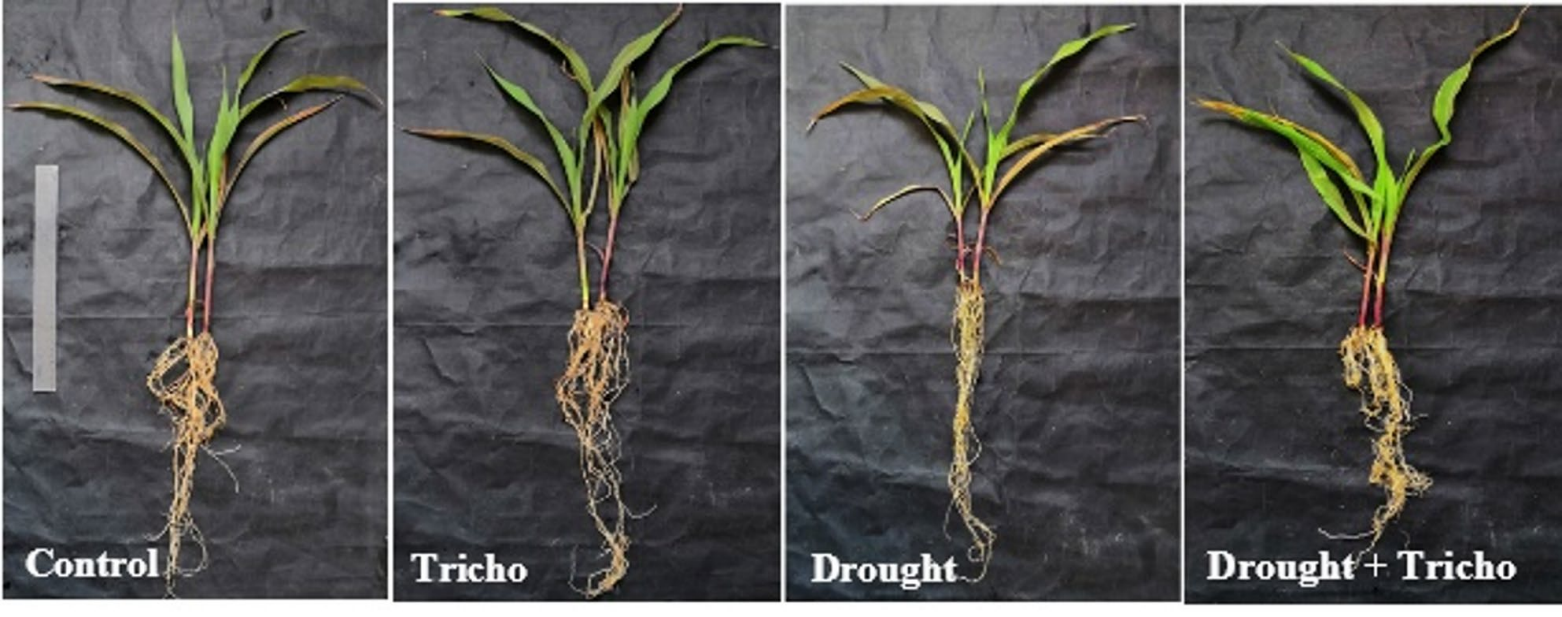
Effect of *T. harzianum* Zag-1 inoculation on the morphological appearance (shoots and roots) of maize (*Zea mays* L.) plants under drought stress (45% FWC)

Drought stress significantly reduced shoot height (17.81%) and root length (15.46%) of maize compared to controls (Table 2 and Fig. 3). Additionally, compared to control plants, 45% FWC declined the fwts (39.44 and 10.29%) and dwts (37.92 and 28.77%) of maize shoots and roots, respectively. Interestingly, *T. harzianum* Zag-1 successfully counteracted this decline by encouraging root and shoot growth even in drought-stressed environments. These results corroborate with Sorahinobar et al. [6] and Abdelhameed and Metwally [13] results in barley and cowpea plants under drought stress. Buragohain et al. [64] and Muhammad et al. [65] stated that plants are severely limited during drought due to decreased soil water supply that causes stomatal closure, decreased cell turgor, decreased CO_2_ assimilation, impaired photosynthetic machinery, and reduced growth and biomass accumulation. In addition to causing osmotic stress and a surge of ROS, it also accelerates leaf senescence and damages proteins, membranes, and nucleic acids [2].

**Table 2:** Impact of *T. harzianum* Zag-1 inoculation on plant growth parameters of maize (*Zea mays* L.) plants under drought stress (45% FWC)

Treatments	Shoot height (cm/plant)	Root length (cm/plant)	Shoot weight (g/plant)		Root weight (g/plant)		No. leaves/ plant	Leaf area (cm^2^)
			fwt	dwt	fwt	dwt		
Control	55.0 ± 2.910a	37.5 ± 1.984b	8.34 ± 0.441b	1.0046 ± 0.053b	3.01 ± 0.158b	0.4132 ± 0.021b	5.0 ± 0.264a	86.10 ± 4.555ab
Tricho	59.1 ± 3.127a	47.5 ± 2.513a	9.79 ± 0.518a	1.2168 ± 0.064a	5.44 ± 0.288a	0.6014 ± 0.031a	5.5 ± 0.291a	100.05 ± 5.294a
Drought (45% FWC)	45.2 ± 2.391c	31.7 ± 1.677b	5.05 ± 0.267c	0.6236 ± 0.032c	2.70 ± 0.143b	0.2943 ± 0.015c	4.0 ± 0.211b	69.22 ± 3.663c
Drought+ Tricho	50.0 ± 2.645ab	36.4 ± 1.926b	6.12 ± 0.324c	0.7737 ± 0.040c	3.11 ± 0.164b	0.3518 ± 0.018bc	5.0 ± 0.264a	79.80 ± 4.222bc

*Data represents the mean of 5 replicates with standard error. Different letters indicate significant differences among treatments using a one-way ANOVA followed by the Duncan’s multiple range test (*p* < 0.05)

However, *T. harzianum* Zag-1 significantly boosted these parameters under both controlled as well as drought stressed conditions (Table 2) compared with the non-inoculated ones. Under stressed conditions, *T. harzianum* Zag-1 increased shoot height, fwt and dwt (10.61, 21.18 and 24.06%, respectively) as well as root length, fwt and dwt (14.82, 15.18 and 19.53%) of the maize plant as compared with controls. *T. harzianum* inoculation lessened the negative impact of drought on the leaf area (15.28%) of stressed maize plants (Table 2). Shah et al. [66] stated that soil or seed applications of *T. harzianum* improved rice growth under 25–75% water regimes, increasing biomass, photosynthesis, and lowering MDA/H_2_O_2_. *T. harzianum* uses a variety of complementary strategies to minimize the drought effects. It frequently improves nutrient mobilization, boosts root development and water intake, and encourages the buildup of osmolytes [23, 61]. Also, Eftekhari et al. [67] stated that *Trichoderma* sp. can alter calcium signaling and phytohormone balances (auxins, ABA, and ethylene). Also, *Trichoderma* sp. simultaneously triggers host defense mechanisms, including antioxidant systems, which result in improved membrane integrity during drought, reduced lipid peroxidation, and quicker ROS scavenging, creating a primed condition that enables a quicker and more efficient reaction to water scarcity [23, 68].

### Photosynthetic pigments

One important early plant response to water constraint is the reduction of photosynthetic pigments [9, 69] as apparent in our results in maize leaves (Table 3). Under drought, maize showed notable decreases in its pigment concentration (Chl a [29.87%], Chl b [19.60%] and carotenoids [18.57%]). According to Shah et al. [1], throughout the vegetative growth and blooming stages of chickpea plants, drought stress can dramatically reduce the Chl. a and Chl. b. Also, *M. parviflora* and bean plants under drought stress showed comparable outcomes [8, 70]. Drought drastically lowers the amount of Chl. in maize leaves as shown in Table 3 by decreasing Chl. biosynthesis (inactivation of biosynthetic enzymes) and increasing Chl. destruction driven by oxidative damage to photosynthetic complexes and chloroplast membranes [2]. Reduced light harvesting and electron transport, stomatal closure-driven CO_2_ restriction, decreased Chl. a and Chl. b, and a general loss in photosynthetic capacity and growth under water scarcity are the overall outcomes as documented in several plants [71–73].

**Table 3:** Impact of *T. harzianum* Zag-1 inoculation on pigment fractions (mg/g fwt) of maize (*Zea mays* L.) plants under drought stress (45% FWC)

Treatments	Chl a (mg/g fwt)	Chl b (mg/g fwt)	Carotenoids (Card) (mg/g fwt)	Total pigment (TP) (mg/g fwt)
Control	1.175 ± 0.0310b	0.459 ± 0.004b	0.689 ± 0.018b	2.323 ± 0.061b
Tricho	1.475 ± 0.039a	0.642 ± 0.0056a	0.965 ± 0.025a	3.082 ± 0.081a
Drought (45% FWC)	0.824 ± 0.021d	0.369 ± 0.0032c	0.561 ± 0.014c	1.754 ± 0.041d
Drought+ Tricho	1.033 ± 0.027c	0.474 ± 0.0041b	0.573 ± 0.015c	2.08 ± 0.056 c

*Values are means of 5 replicates with standard error. Different letters indicate significant differences among treatments using a one-way ANOVA followed by the Duncan’s multiple range test (*p* < 0.05)

It’s interesting to note that, in comparison to drought-stressed plants, those inoculated with *T. harzianum* Zag-1 showed a notable rise in Chl. a (25.36%), Chl. b (28.45%) and carotenoids (2.13%) contents (Table 3). Sabzi-Nojadeh et al. [74] reported that *T. harzianum* preserved photosynthetic efficiency and ROS homeostasis by raising carotenoid and Chl. levels in *Satureja hortensis* during salt stress. Carotenoids function as antioxidants and lessen lipid peroxidation, while Chl. preservation guarantees ongoing light harvesting under stress [22]. *Trichoderma* spp. operates on several levels to reduce Chl. loss caused by drought [6] by increasing host antioxidant systems that lessen ROS-mediated chloroplast damage, slow down Chl. degradation, and root and rhizosphere interactions that enhance water uptake and nutrient availability (including Fe and N_2_), which supports Chl. biosynthesis [61, 67].

### Water relations

The RWC of maize leaves, a crucial measure of water balance and turgor maintenance which represents the hydration plant’s level, significantly decreases during drought stress. Table 4 showed that RWC (7.90%) exhibits a subsequent drop in maize leaves under drought stress as compared to the control plants. In contrast to the control, WSD (39.63%) rose during drought stress (Table 4). Due to the detrimental effects of drought on stomatal opening and closure processing, drought stress alters plant water relations by lowering water availability, lowering leaf water potential, transpiration rate, and raising leaf temperature [75–77]. By improving water uptake and retention in plant tissues, *T. harzianum* has been demonstrated to mitigate this loss in water-deficit situations. According to the current results, maize treated with *T. harzianum* Zag-1 showed significantly greater RWC (11.15%) under drought circumstances as compared to non-inoculated controls **(**Table 4**)**. These findings are supported by Aljeddani et al. [78], who reported that *T. harzianum* boosted RWC in drought-exposed bread wheat plants through improved root growth and greater expression of aquaporin, allowing for more effective water absorption. Additionally, Racić et al. [79]. revealed that tomato plants treated with *T. brevicompactum* had a 17% greater RWC than that of untreated plants under drought conditions. Additionally, under extreme drought stress, *T. atroviride* ID20G increased RWC in maize seedlings by up to 12%, enabling it to absorb more water from the rhizosphere and enhancing the plant’s resistance to drought stress [80]. Additionally, by improving rhizosphere colonization and extracellular polysaccharide synthesis, *Trichoderma* spp. increase soil moisture availability and soil water-holding capacity [23]. Overall, *T. harzianum’s* critical role in maintaining plant water relations, postponing dehydration, and guaranteeing physiological activity under water shortage is highlighted by the maintenance of greater RWC in *T. harzianum*-treated maize plants under drought stress [61, 81].

**Table 4:** Impact of *T. harzianum* Zag-1 inoculation on water status (RWC and WSD) and membrane traits (MSI, MI and El (%) of maize (*Zea mays* L.) plants under drought stress (45% FWC)

Treatments	Relative water content (RWC) (%)	Water saturation deficient (WSD) (%)	Membrane stability index (MSI) (%)	Membrane Injury (MI) (%)	EL (%)
Control	83.34 ± 2.205ab	16.65 ± 0.440b	83.61 ± 2.212a	0c	16.38 ± 0.433c
Tricho	87.78 ± 2.322a	12.21 ± 0.323c	85.41 ± 2.259a	0c	14.58 ± 0.385d
Drought (45% FWC)	76.75 ± 2.083b	23.25 ± 0.562a	75.75 ± 2.004b	9.40 ± 0.248a	24.25 ± 0.641a
Drought+ Tricho	85.31 ± 2.280ab	14.68 ± 0.415b	79.62 ± 2.106ab	6.78 ± 0.179b	20.37 ± 0.539b

*Data presented represent the mean of 5 replicates with standard error. Different letters indicate significant differences among treatments using a one-way ANOVA followed by the Duncan’s multiple range test (*p* < 0.05)

### Stress markers (EL, MI, MDA, and H_2_O_2_)

**Fig. 4:**
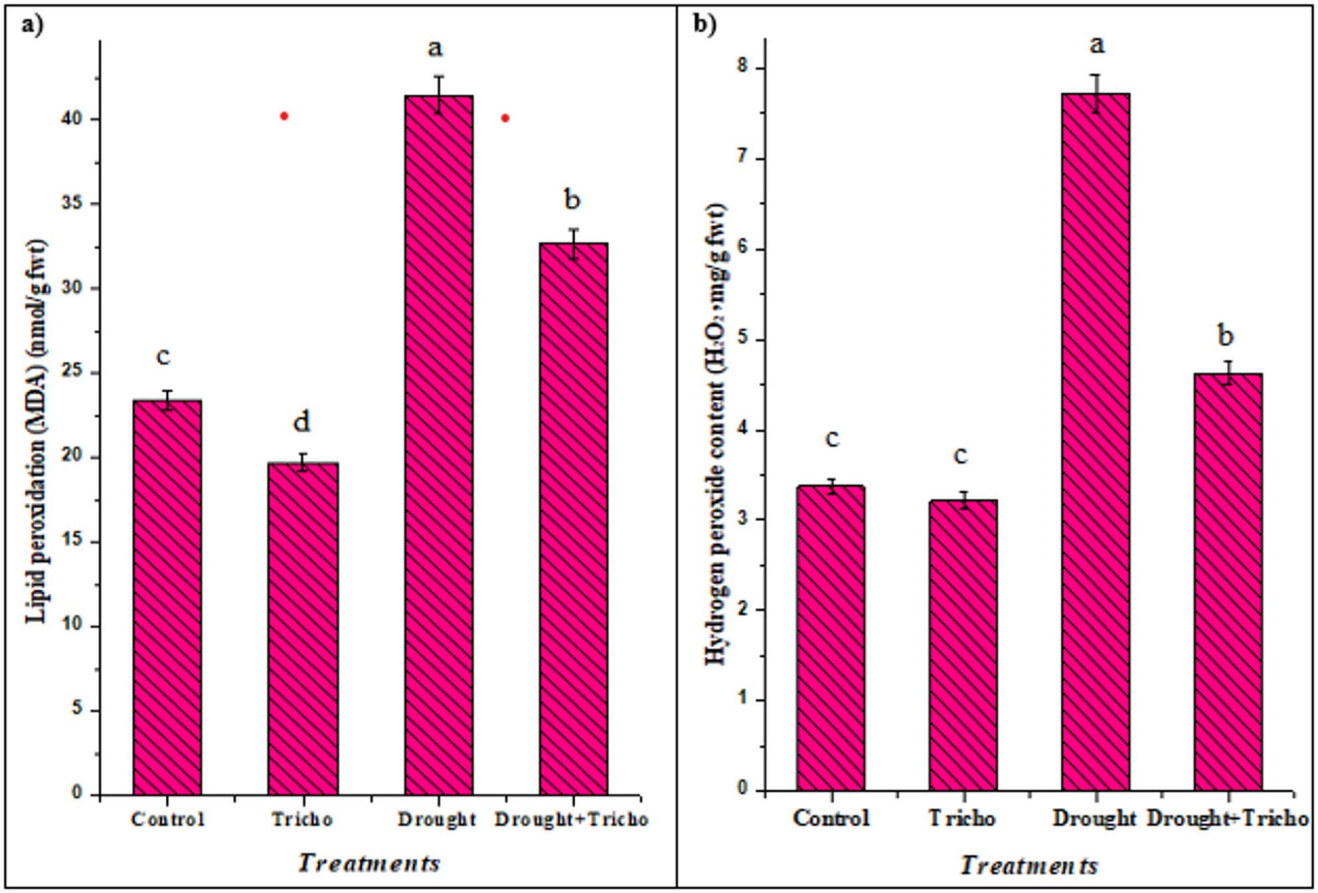
Impact of *T. harzianum* Zag-1 inoculation on the oxidative stress markers (**a**: lipid peroxidation [MDA, nmol/g fwt] and **b**: Hydrogen peroxide [H_2_O_2_, mg/g fwt] contents) of maize (*Zea mays* L.) plants under drought stress (45% FWC). Data are means ± standard errors, columns followed by the same letters do not differ statistically by Duncan test, *p* < 0.05

In maize, drought (45% FWC) caused a clear rise in oxidative stress markers in their leaves, represented by EL (48.04%), MDA (77.21%), MI (9.40%), and H_2_O_2_ (128.94%), all increased in drought-stressed maize plants compared with well-watered controls (*p* < 0.05) (Table 4 and Fig. 4). Conversely, *T. harzianum*-inoculated plants showed significantly lower EL (16.00%), MDA (21.16%), MI (6.78%), and H_2_O_2_ (40.10%) than the non-inoculated ones under 45% FWC— typically a partial restoration toward control values (MI, EL, and MDA reduced, and H_2_O_2_ accumulation attenuated) (Table 4 and Fig. 4). These protective changes were accompanied by higher activities of key antioxidant enzymes (e.g., PPO, POD, and APX, as appeared later in Fig. 6) in inoculated plants. The increase of these stress markers under 45% FWC confirms that drought provokes membrane damage and ROS accumulation in maize leaves. These results agree with Abdelhameed et al. [8] and Abdelhameed and Metwally [13] results in malva and cowpea under drought as ROS diffuse *via* membrane, damaging cells far from their production origin [9, 82]. In plant cells, ROS may associate with superoxide radicals to create hydroxyl radicals, which can cause cell death as well as protein degradation [83]. Also, upsurges in ROS can cause lipid peroxidation of membranes’ polyunsaturated component, which will impact the membrane fluidity and functionality, leading to more EL and MDA production (Table 4 and Fig. 4). *T. harzianum* inoculation markedly limited this damage under water deficit, most likely by inducing antioxidant defenses, osmolytes, and improving plant growth and water relations, which together reduce ROS production and lipid peroxidation [6, 77]. As further evidence, *T. harzianum* inoculation decreased H_2_O_2_ levels in cucurbits under salt stress, effectively lowering oxidative damage [84]. Metwally et al. [15] and Metwally and Soliman [85] revealed that under pathogen infection and salt, barley and tomatoes plants inoculated with *T. asperellum* and *T. viride* have lower H_2_O_2_ levels, suggesting that they are effective in H_2_O_2_ scavenging and protecting the plant from stress effect.

### Osmoprotectants accumulation

**Fig. 5:**
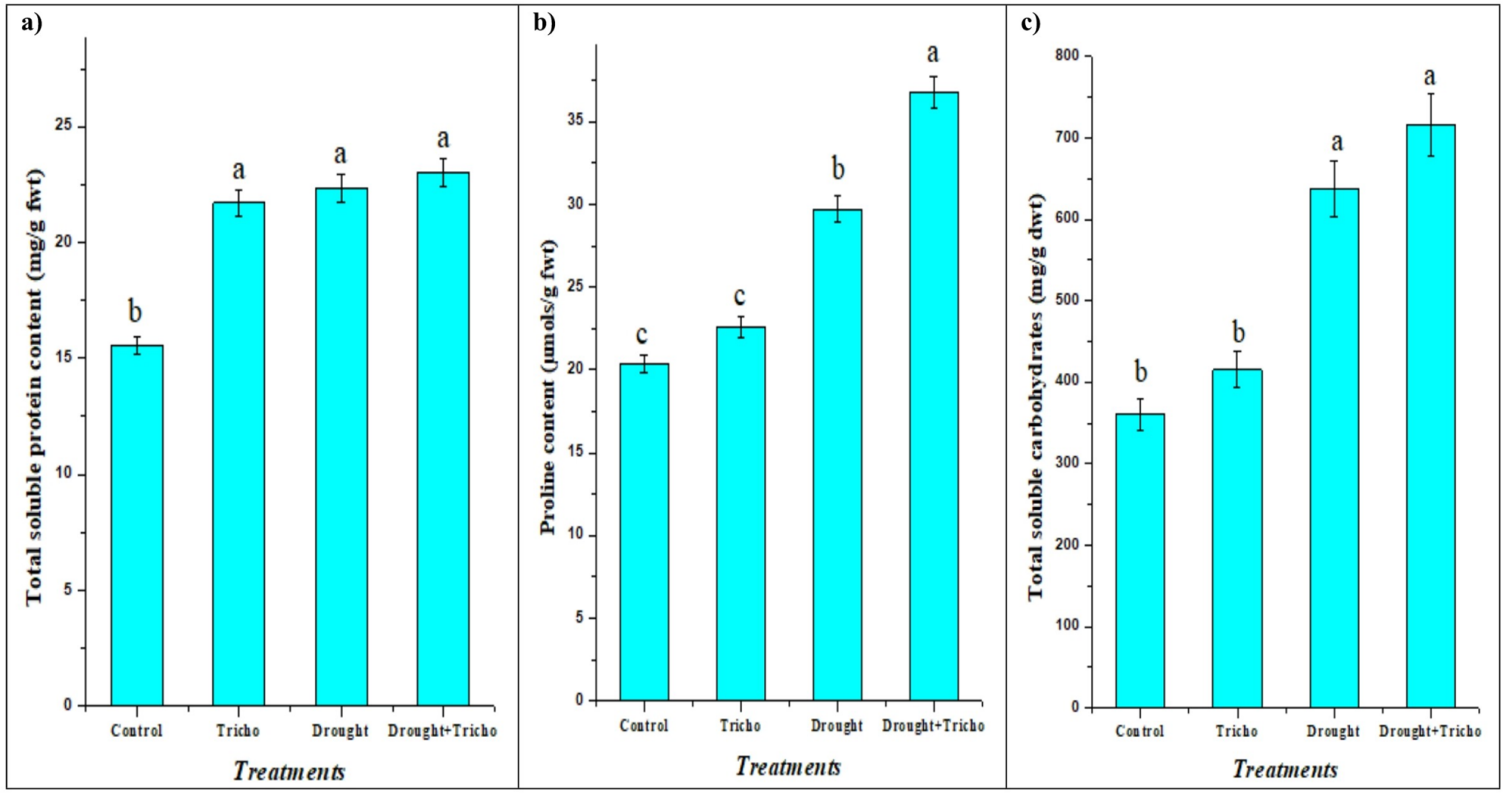
Impact of *T. harzianum* Zag-1 inoculation on osmoregulatory substances: **a**) total soluble protein content (mg/g fwt), **b**) proline content (µmols/g fwt) and **c**) total soluble carbohydrates (mg/g dwt) of maize (*Zea mays* L.) plants under drought stress (45% FWC). Data are means ± standard errors, columns followed by the same letters do not differ statistically by Duncan test, *p* < 0.05

Consequently of plants suffering from osmotic imbalance, oxidative stress, and disturbance of cellular homeostasis due to water deficit stress as previously mentioned in Table 4 and Fig. 4, plants have evolved several mechanisms to deal with these negative effects. One of these adaptive mechanisms is osmoprotectants accumulation, or osmolytes, which are low-molecular-weight organic compounds like proline, soluble sugars, and amino acids [86–88]. Our results showed that *T. harzianum* Zag-1 and drought changed the cellular osmolytes content (Fig. 5). The physiological changes caused by drought stress were evident in the significantly greater amounts of proline (45.93%), soluble sugars (76.67%), and proteins (43.73%) in maize plants compared to well-irrigated conditions.

Osmolytes accumulation has been widely linked to improved drought tolerance in a number of crops, including soybean, barley, malva, maize and rice [3, 6, 8, 29, 89]. Eftekhari et al. [67] and Ahmed et al. [89] stated that, under drought conditions, these osmoprotectants help cells maintain turgor pressure and water uptake by assisting in osmotic adjustment. In addition to their osmoprotective role, osmolytes scavenge ROS, stabilize proteins and membranes, and shield cellular structures from denaturation driven by dehydration. Among these, proline aids in signaling during stress recovery, redox buffering, and subcellular structural stabilization [90]. Likewise, glycine betaine improves the stability of enzymes during dehydration and maintains the integrity of photosynthetic machinery [13]. Under controlled and drought-induced circumstances, *T. harzianum* treatment effectively raised the levels of protein, proline, and soluble carbohydrates when compared to non-inoculated plants. When compared to the non-treated ones, *T. harzianum* exhibited a 3.04, 23.78, 12.28% increase in their contents (Fig. 5), which ultimately assisted the inoculated plants cope with the drought stress more effectively. Recent research shows that in addition to plants, PGPM that promote plant growth, such rhizobacteria, arbuscular mycorrhiza and *Trichoderma* spp., can increase osmolytes production in host plants, increasing their resistance to different stresses [6, 15, 89]. According to Metwally et al. [15] and Aalipour et al. [91], *T. asperellum* and PGPR improved the osmolytes in barley and *Arizona cypress* plants. This, in turn, raised cell turgor pressure, safeguarded the cells’ water status, and maintained the membranes [92]. The low level of peroxidation (less MDA) of cell membranes (Fig. 4a) demonstrates this improvement.

### Antioxidant enzymes

**Fig. 6:**
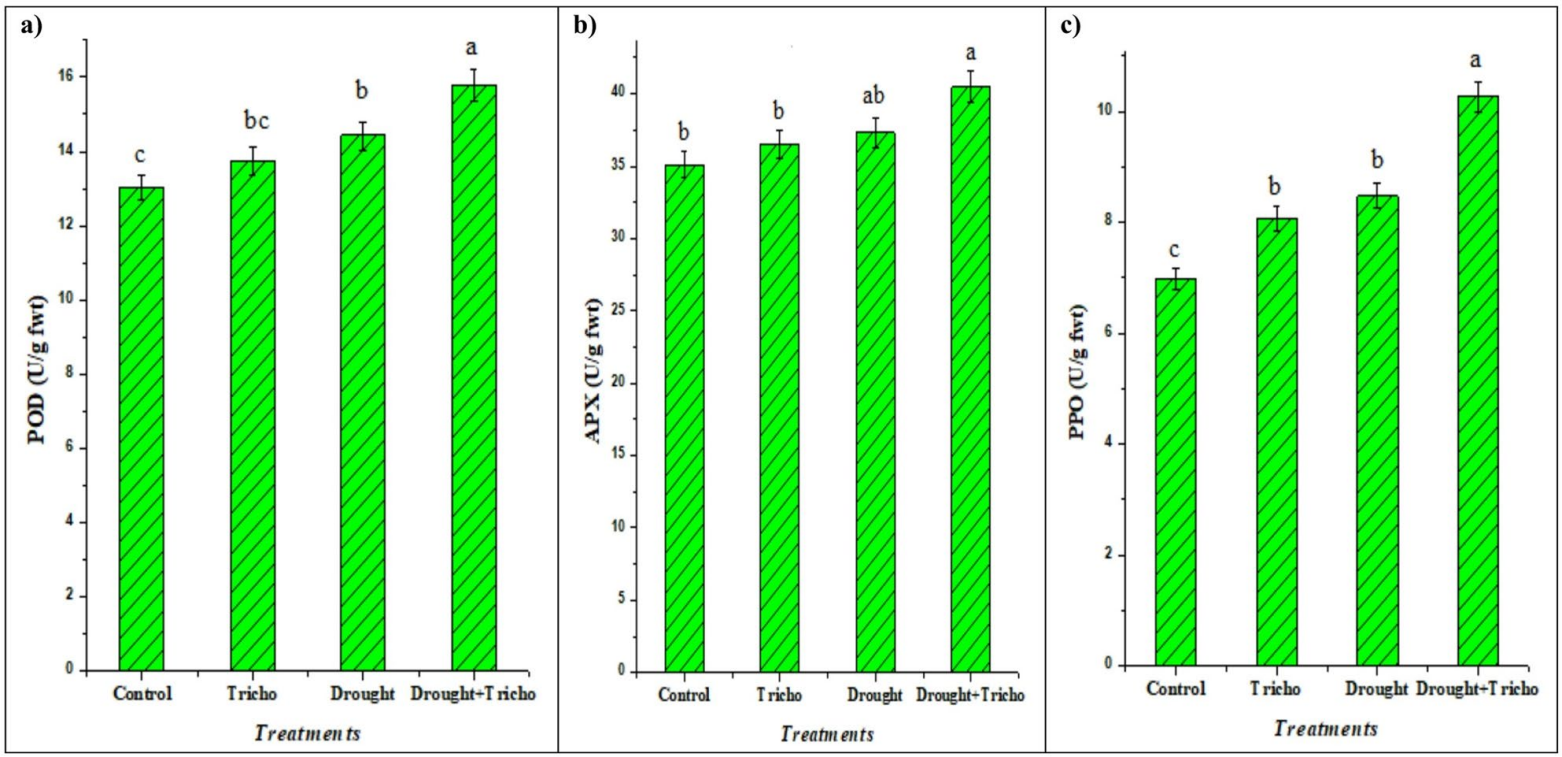
Impacts of *T. harzianum* Zag-1 inoculation on antioxidant enzymes: (**a**) peroxidase (POD), (**b**) ascorbate peroxidase (APX) and (**c**) polyphenol oxidase (PPO) in the shoots of maize (*Zea mays* L.) plants under drought stress (45% FWC). *Data presented represent the mean of 5 replicates with standard error. Different letters indicate significant differences among treatments using a one-way ANOVA followed by the Duncan’s multiple range test (*p* < 0.05)

Antioxidant enzymes such as PPO, POD, and APX are another adaptive mechanism that converts ROS into less harmful compounds that inhibit oxidative damage and promotes photosynthetic efficiency [67, 93]. Results in Fig. 6 were related to the effect of drought stress and *T. harzianum* application on APX, POD, and PPO activities in maize plant leaves. Under drought stress (45% FWC), maize leaves showed significant induction (ANOVA, *p* < 0.05) of antioxidant enzymes relative to well-watered controls. It increased the APX, POD, and PPO by 6.18, 10.82 and 21.35% relative to control (Fig. 6). Interestingly, *T. harzianum*-inoculated plants exhibited a further increase in their activities compared with non-inoculated plants either under controlled (4.02, 5.46 and 15.52%) or drought-stressed conditions (8.53, 9.42 and 21.20%) (Fig. 6).

**Fig. 7:**
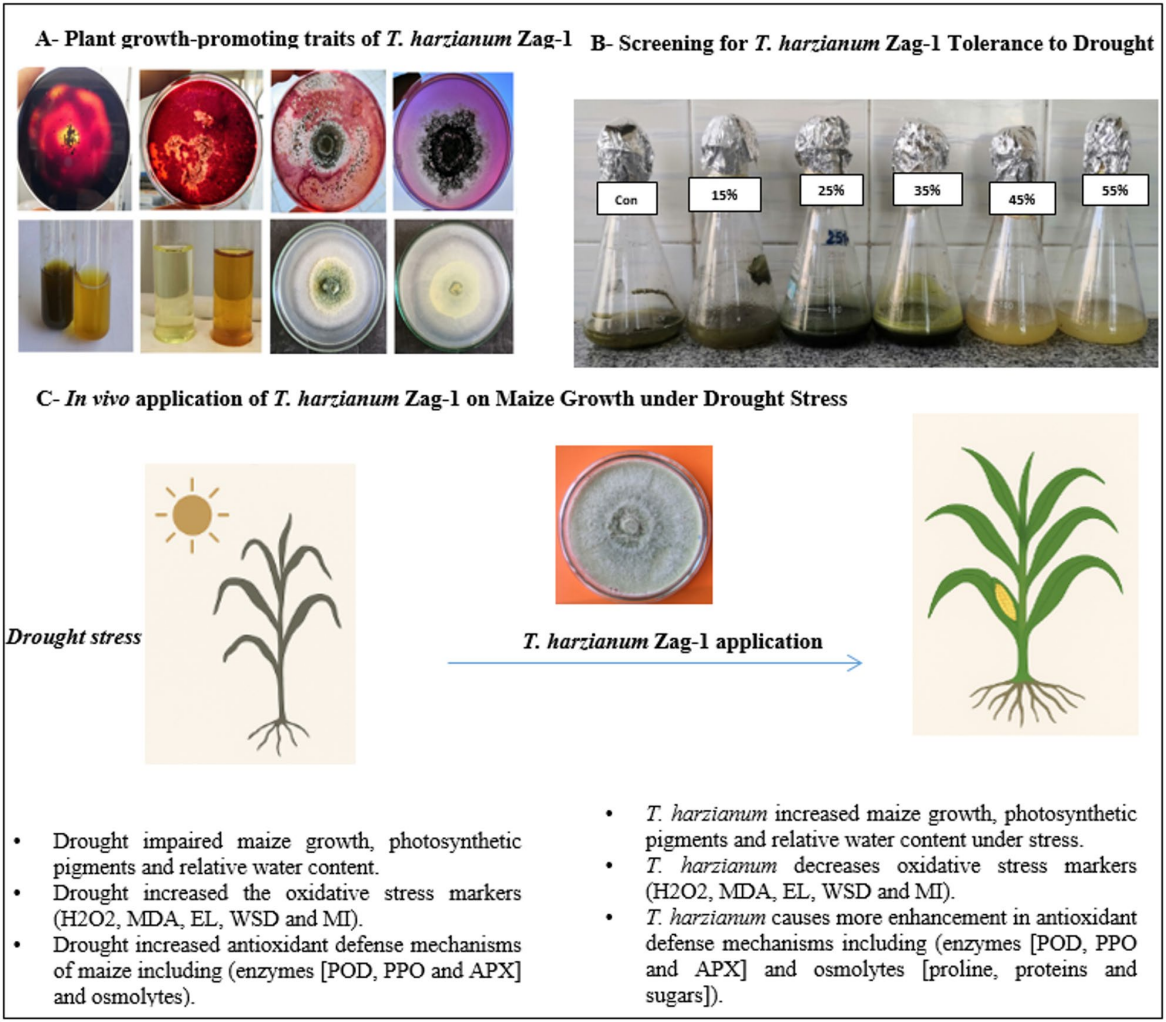
Schematic Overview of *T. harzianum* Zag-1 for plant growth attributes and drought stress mitigation in maize plants APX: Ascorbate peroxidase, EL: Electrolyte leakage, H_2_O_2_: Hydrogen peroxide, MDA: Malondialdehyde, MI: Membrane Injury, POD: Peroxidase, PPO: Polyphenol oxidase, WSD: Water saturation deficit

The enhanced enzyme activities in inoculated plants were accompanied by lower markers of oxidative damage (reduced EL, MI, H_2_O_2_, and MDA) reported in parallel measurements as previously mentioned in Table 4 and Fig. 4, indicating more effective ROS detoxification in *T. harzianum*-treated maize under 45% FWC, and support a protective, enzyme-mediated detoxification mechanism. APX and POD directly scavenge H_2_O_2_ while PPO contributes to phenolic-based antioxidant capacity, together lowering ROS and lipid peroxidation [94]. The findings were confirmed by Sorahinobar et al. [6], Eftekhari et al. [67] and Metwally and Soliman [85], who found that barley, maize and tomatoes plants inoculated with *T. harzianum* and *T. viride* had higher levels of antioxidant enzymes than the untreated control. Also, *B. velezensis* RaSh2 and *T. harzianum* raised SOD, APX, and POD in *Vigna unguiculata* L. and *Brassica juncea* L. gives these plants resistance to drought and salt stresses according to Abdelhameed and Metwally [13] and Ahmad et al. [94]. Shah et al. [66] and Metwally and Soliman [85] reported increases in POD and APX activities in *Trichoderma*-treated rice and tomatoes under saline conditions. *Trichoderma* likely acts through a combination of root colonization, hormonal modulation, and priming of defense gene expression to upregulate antioxidant enzymes, thereby stabilizing membranes and preserving physiological function under 45% FWC [23, 63, 81]. Therefore, increased antioxidant enzyme activities in maize leaves under drought stress (45% FWC) have been linked to greater drought tolerance. A schematic overview of *T. harzianum* for plant growth attributes and drought stress mitigation in maize plants was represented in Fig. 7.

## Conclusion

Maize plants are severely harmed by drought stress, which results in a considerable decrease in morphological characteristics, chlorophyll content, and RWC as well as an increase in H_2_O_2_ and MDA buildup. These negative effects were successfully lessened by *T. harzianum* Zag-1 application which decreased oxidative damage and restored redox equilibrium, assisting in osmotic adjustment and cellular structure protection. These highlight the potential significance of *T. harzianum* Zag-1 in climate-smart agriculture by showing that it improves crop resilience under drought conditions. Zag-1 is a sustainable biological input that can supplement traditional management techniques by promoting plant stress tolerance and sustaining development under water scarcity. Its use could improve crop performance in drought-prone areas, offering a workable and eco-friendly approach to sustainable crop management in the face of shifting climate circumstances.

## Data Availability

Data will be made available on request.

## Abbreviations

APX: Ascorbate peroxidase
CMC: Carboxymethyl cellulose
DWT: Dry weight
EC: Electrical conductivity
EL: Electrolyte leakage
FWT: Fresh weight
FWC: Field water capacity
GB: Glycine betaine
IAA: Indole-3-acetic acid
MDA: Malondialdehyde
MI: Membrane Injury
MSI: Membrane stability index
POD: Peroxidase
PPO: Polyphenol oxidase
PGP: Plant Growth-Promoting
PEG: Polyethylene glycol
RWC: Relative water content
SE: Standard error
ROS: Reactive oxygen species
TBA: Thiobarbituric acid
WSD: Water saturation deficit
